# Expression of the potential therapeutic target CXXC5 in primary acute myeloid leukemia cells - high expression is associated with adverse prognosis as well as altered intracellular signaling and transcriptional regulation

**DOI:** 10.18632/oncotarget.3056

**Published:** 2014-12-26

**Authors:** Øystein Bruserud, Håkon Reikvam, Hanne Fredly, Jørn Skavland, Karen-Marie Hagen, Tuyen Thy van Hoang, Annette K. Brenner, Amir Kadi, Audrey Astori, Bjørn Tore Gjertsen, Frederic Pendino

**Affiliations:** ^1^ Section for Hematology, Department of Clinical Science, University of Bergen, Norway; ^2^ Department of Medicine, Haukeland University Hospital, Bergen, Norway; ^3^ Department of Molecular Biology, University of Bergen, Bergen, Norway; ^4^ Inserm, U1016, Institut Cochin, F-75014, Paris, France; ^5^ CNRS, UMR8104, F-75014, Paris, France; ^6^ Université Paris Descartes, Sorbonne Paris Cité, Paris, France

**Keywords:** Acute myeloid leukemia, CXXC5, transcription, cytokines, all-trans retinoic acid

## Abstract

The CXXC5 gene encodes a transcriptional activator with a zinc-finger domain, and high expression in human acute myeloid leukemia (AML) cells is associated with adverse prognosis. We now characterized the biological context of CXXC5 expression in primary human AML cells. The global gene expression profile of AML cells derived from 48 consecutive patients was analyzed; cells with high and low CXXC5 expression then showed major differences with regard to extracellular communication and intracellular signaling. We observed significant differences in the phosphorylation status of several intracellular signaling mediators (CREB, PDK1, SRC, STAT1, p38, STAT3, rpS6) that are important for PI3K-Akt-mTOR signaling and/or transcriptional regulation. High CXXC5 expression was also associated with high mRNA expression of several stem cell-associated transcriptional regulators, the strongest associations being with WT1, GATA2, RUNX1, LYL1, DNMT3, SPI1, and MYB. Finally, CXXC5 knockdown in human AML cell lines caused significantly increased expression of the potential tumor suppressor gene TSC22 and genes encoding the growth factor receptor KIT, the cytokine Angiopoietin 1 and the selenium-containing glycoprotein Selenoprotein P. Thus, high CXXC5 expression seems to affect several steps in human leukemogenesis, including intracellular events as well as extracellular communication.

## INTRODUCTION

CXXC5 is a retinoid-responsive gene localized to the 5q31.3 chromosomal region [1] and encoding a retinoid-inducible nuclear factor (RINF) [2] that is a protein containing a CXXC-type zinc-finger domain and acting as a transcription regulator [3]. Expression studies as well as gene silencing experiments suggest that CXXC5 is important in normal myelopoiesis [2] and for differentiation of endothelial cells [3]. Furthermore, we recently described that CXXC5 is expressed in primary acute myeloid leukemia (AML) cells; this expression shows a wide variation between patients and high levels are associated with an adverse prognosis and resistance to chemotherapy-induced apoptosis [4]. Another study recently confirmed our observations and CXXC5 expression was then of independent prognostic significance in multivariate analyses after adjustment for age, white blood cell count, cytogenetic risk group, *FLT3*-ITD status, biallelic *CEBPA* mutations, as well as mutations of *NPM1*, *DNMT3A* and *ASXL1* [5]. Based on these observations we suggest that CXXC5 should be considered as a possible therapeutic target in human AML. However, more detailed preclinical evaluation of CXXC5 as a possible therapeutic target is needed. In the present study we characterized the biological context of high CXXC5 expression and effects of CXXC5 knockdown in human AML cells.

## MATERIAL AND METHODS

### AML patients and preparation of primary AML cells

The study was approved by the Regional Ethics Committee III, University of Bergen, Norway). Samples were collected after written informed consent, and we included consecutive and thereby unselected patients with high peripheral blood blast counts (>7 × 10^9^/L) (Table 1). These selections of patients as well as the analysis of FLT3 and NPM1 mutations have been described previously [6, 7]. AML cells were isolated by density gradient separation alone (Lymphoprep, Axis-Shield, Oslo, Norway) and contained at least 95% leukemic blasts. The cells were stored in liquid nitrogen until used in the experiments [6]. CXXC5 expression was determined by PCR analysis for a cohort of 67 consecutive patients and global gene expression profiles were analysed in a second cohort of 48 consecutive patients; there was an overlap of 24 patients between the cohorts (see later, Suppl. Fig. 1).

**Table 1:** Clinical and biological characteristics of the AML patients included in the study

Parameter	All 67 patients	Low CXXC5 expression 34 patients	High CXXC5 expression 33 patients
Age (years; median/range)	64 years (27-88 years)	61 years (29-88 years)	70 years (27-83 years)
Gender distribution (female/male)	30/37	16/18	13/20
Percentage of patients with:			
AML secondary to chemotherapy	8%	12%	3%
AML secondary to myeloid malignancies (MDS, chronic myeloid neoplasia)	24%	18%	30%
*de novo* AML	68%	70%	67%
Relapse at the time of examination	21%	15%	27%
FAB classification			
M0/M1	36%	24%	48%
M2	26%	27%	26%
M4/M5	38%	49%	48%
Expression of CD34 (>20% positive AML cells)	54%	54%	54%
Cytogenetic abnormalities			
Normal	59%	54%	64%
Good	8%	17%	0
Intermediate	9%	4%	12%
Adverse	27%	25%	30%
Flt3 internal tandem duplication (ITD)	32%	29%	37%
NPM-1 mutations	45%	45%	45%

Favourable cytogenetic abnormalities include inv(16)/t(16;16) and t(8;21); adverse include multiple (≥ 3 abnormalities), t(3:3), del7 and del5.

### AML cell lines

Human leukemic cell lines were purchased from DSMZ (MV4-11; Braunschweig, Germany) and from the American Type Culture Collection (K562; Molsheim, France). UT7 5.3 cells were kindly provided by Isabelle Dusanter-Fourt (Cochin Institute, Paris, France). K562 and MV4-11 were cultured in RPMI 1640 medium supplemented with 10% fetal calf serum (FCS), 2 mM L-Glutamine, 50 U/ml penicillin G and 50 μg/ml streptomycin (Life Technologies, Saint-Aubin, France). UT7 5.3 cells were cultured in minimum essential medium (MEM) α medium containing 10% of FCS, 2 mM L-Glutamine, 50 U/ml penicillin G and 50 μg/ml streptomycin (Life Technologies, Saint-Aubin, France) and 2,5 ng/μl of GM-CSF (Myltenyi Biotech, France).

### RNA purification and quantitative RT-PCR analysis of CXXC5 messenger RNA expression

The methods for purification of total RNA, complementary DNA synthesis and quantitative PCRs (qPCR) have been described in detail previously [4]. Relative messenger RNA (mRNA) expression was normalized to ribosomal protein P2 (RPLP2) gene expression in a two-colour duplex reaction.

### RNA preparation, labeling and microarray hybridization for primary human AML cells

Microarray analyses were performed using Illumina iScan Reader based on fluorescent detection of biotin-labeled cRNA. Total RNA (300 ng) from each sample was reversely transcribed, amplified and Biotin-16-UTP-labelled using Illumina TotalPrep RNA Amplification Kit (Life Technologies, Foster City, CA, USA). Amount and quality of biotin-labeled cRNA was controlled by NanoDrop spectrophotometer and by Agilent 2100 Bioanalyser (Agilent Technologies, Santa Clara, CA, USA). Biotin-labeled cRNA (750 ng) was hybridized to HumanHT-12V4 Expression BeadChip according to the manufacturer's instructions. The HumanHT-12V4 BeadChip targets 47231 probe was based primarily on genes in the National Center for Biotechnology Information RefSeq database (Release 38; ftp://ftp.cbi.edu.cn/pub/database/refseq/release/release-notes/archive/RefSeq-release38.txt).

### *In vitro* culture of primary human AML cells

#### Drugs

Lenalidomide (Selleck Chemicals, Munich, Germany) was used at 0.5 μM. The mTOR inhibitor rapamycin was purchased from LC Laboratories (Woburn, MA, USA) and the pan-PI3K inhibitor GDC-0941 from Axon Mechen (BV, Groningen, the Netherlands); both were used at 1.0 μM. 17-dimethylaminoethylamino-17-demethoxygeldanamycin (17-DMAG) (Infinity Pharmaceuticals, Cambridge, MA, USA) was used at 1.0 μM. Bortezomib was purchased from Jansen-Cilag (Beerse, Belgium) and used at 25 nM. Ingenol-3 angelate (PEP005) was supplied by Peplin Ltd (Brisbane, Australia) and used at 20 nM. Protein kinase inhibitors were all purchased from Biaffin GmbH (Kassel, Germany); PD98059 was used at 20 μM whereas SB202190 and SP600125 were used at 1 μM. All drug solutions except GDC-0941 were prepared in dimethylsulphoxide (DMSO) or ethanol; pilot experiments demonstrated that DMSO or ethanol at the final concentrations used in the experiments did not affect AML cells.

#### Cell culture

Cells were cultured in StemSpan serum-free medium supplemented with 100 μg/ml of gentamicin (Stem Cell Technologies Inc, Vancouver, BC, Canada) [8]. AML cells (1 × 10^6^ cells/ml) were cultured at 37ºC in a humidified atmosphere of 5% CO_2_. Cytokine levels in supernatants were analysed by Luminex methodology after 48 hours of culture (Bio-Plex human cytokine group, Bio-Rad, Oslo, Norway) [7-9]. AML cell viability/apoptosis was analysed by flow cytometry [10, 11].

### Intracellular protein phosphorylation

Protein phosphorylation was analyzed by flow cytometry as described in detail previously [12-14].

### CXXC5 knockdown and global gene expression analyses of AML cell lines

#### CXXC5 knockdown

K562 and UT7.3 cells were transduced with the pTRIP lentiviral vector that drives the constitutive expression of GFP (Green Fluorescent Protein) for cell sorting, and either a short-hairpin RNA [shRNA] targeting RINF sequence (shRNA-RINF) or a non-relevant sequence (non-target-shRNA control) as described in detail previously [1]. K562 and MV4-11 were also transduced with the previously described pLKO.1/shRNA-RINF and pLKO.1/shRNA-nontarget that drives the expression the puromycine resistant gene for the selection of the cells [1]. Lentiviral plasmids (pLKO.1/short hairpin RNA [shRNA]/RINF) targeting RINF expression were purchased from Sigma-Aldrich (MISSION shRNA Bacterial Glycerol Stock), and control vectors (pLKO.1/TRC and pLKO.1/shRNA/scramble controls) were kindly provided by David Root and David M. Sabatini (both from Massachusetts Institute of Technology, Cambridge, MA; Addgene plasmids 10879 and 1864). Briefly, production of lentiviral particles were performed by transient cotransfection (with Fugene HD) of HEK 293T cells with the second generation packaging system (e.g., packaging plasmid psPAX2 and envelope plasmid pMD2.G) developed by D. Trono's laboratory (Ecole Polytechnique Fédérale de Lausanne, Lausanne, Switzerland; Addgene plasmids 12260 and 12259). Viral supernatants were harvested and filtered 2 days posttransfection and then applied to growing cells for spin infection (2400 rpm for 1 hour at room temperature), which was carried out in presence of 5 μg/mL proteamine sulfate. Two days after infection, AML cells were selected for at least 2 days with puromycine (Sigma-Aldrich) at 1 μg/mL.

Our methods for knockdown, verification of the knockdown at the protein and mRNA level, and analysis of the effects of knockdown on proliferation and apoptosis have been described in detail in our previous publication [4]. The knockdown alone did not affect proliferation or viability of the AML cell lines, and the efficiency of the knockdown was verified both at the mRNA and protein level for each cell line in every experiment.

#### Analysis of global gene expression profiles

After validation of RNA quality with Bioanalyzer 2100 (using Agilent RNA6000 nano chip kit), 400 ng of total RNA was reverse transcribed following the Genechip WT plus Reagent kit (Affymetrix). Briefly, the resulting double strand cDNA was used for *in vitro* transcription with T7 RNA pol. After purification, 15 μg of cRNA was used for reverse transcription with random primers. The cDNA obtained was then purified and fragmented. After control of fragmentation using Bioanalyzer 2100, cDNA was end labeled with biotin using Terminal Transferase (using the WT terminal labeling kit of Affymetrix). cDNA was then hybridized to GeneChip® Human Transcriptome Analysis 2.0 (Affymetrix) at 45°C for 17 hours. After overnight hybridization, chips were washed on the fluidic station FS450 following specific protocols (Affymetrix) and scanned using the GCS3000 7G. The image was then analyzed with Expression Console software (Affymetrix) to obtain raw data (cel files) and metrics for Quality Controls. Minimum information about a microarray experiment (MIAME)–compliant documentation of the microarray experiments have been deposited in Array Express at the European Bioinformatics Institute (http://www.ebi.ac.uk/arrayexpress). The Affymetrix HTA2 dataset analysis was performed by GenoSplice technology (www.genosplice.com). Data were normalized using quantile normalization. Background corrections were made with antigenomic probes and probes were selected according to their %GC, cross-hybridization status and potential overlap with repeat region as previously described [15, 16]. Only probes targeting exons and exon-exon junctions annotated from FAST DB^®^ transcripts (release fastdb_2014_1) were selected [17, 18]. Only genes expressed in at least one compared condition were analyzed. To be considered to be expressed, the DABG P-value had to be ≤0.05 for at least half of the gene probes. We performed an unpaired Student's t-test to compare gene intensities between shRINF and shCTRL cells. Genes were considered significantly regulated when fold-change was ≥1.5, a fold-change noticed for CXXC5 genes for which we have validated the knock-down by q-RT-PCR and western-blot analysis.

### Analyses of the data

For statistical comparisons between different groups we used the Mann-Whitney U-test, for analyses of paired observations the Wilcoxon's signed rank test was used and for correlation analyses we used the Pearson's correlation test. Differences were regarded as significant when p<0.05. Bioinformatical analyses of gene expression data were performed using the J-Express (MolMine AS, Bergen, Norway); whereas our analysis of protein functions was based on the Panther (http://www.pantherdb.org/) and Reactome (http://www.reactome.org/) databases.

## RESULTS

### Global gene expression profiles differ between AML cells with high and low CXXC5 expression

CXXC5 expression was analyzed by PCR for a total of 67 patients who were included in the study (Table 1). When comparing the 34 patients with low and the 33 patients with high CXXC5 expression we confirmed that monocytic differentiation of the leukemic cells (FAB-M4/M5) was associated with low expression compared with neutrophilic differentiation (FAB-M2) [4], whereas CXXC5^HIGH^ and CXXC5^LOW^ patients did not differ significantly for any other parameter, including the percentage of patients with at least one risk factor for chemoresistance (i.e. secondary leukemia, relapsed disease, adverse cytogenetic abnormalities or Flt3-ITD). Similar results were observed when comparing CXXC5^HIGH^ and CXXC5^LOW^ patients in the microarray cohort (n=48) (data not shown). Both cohorts included consecutive and thereby unselected patients and 24 patients were included in both cohorts; a strong correlation was then observed for these patients when comparing CXXC5 levels determined by PCR and microarray analyses (Suppl. Fig. 1; r=0.7665 p<0.0001, Pearson's correlation test).

We did a Gene Set Enrichment Analysis (GSEA) where we compared the 15 patients with the highest and the 15 patients with the lowest CXXC5 expression, and we then identified 38 GO-terms with a false discovery rate (RDR) <1.0. All GO-terms were enriched in the CXXC5^LOW^ group, and we identified 571 genes that belonged to the leading edge for at least one of these 38 terms. When these 571 genes were used in a hierarchical clustering analysis we identified two main subsets corresponding to the original CXXC5^LOW^ and CXXC5^HIGH^ patient subsets (Suppl. Fig. 2).

The 38 GO-annotations identified above (Suppl. Fig. 2) could be classified into three main groups; extracellular communication (9 annotations), intracellular signaling and trafficking (13 annotations), and a third heterogeneous group including regulation of cell shape, inflammatory and metabolic responses (16 annotations) (Supplementary Table 2). Firstly, CXXC5^HIGH^ and CXXC5^LOW^ patients differed with regard to extracellular communication including cytokine production (IL6, TNFα), integrin-mediated signaling and expression of immunoglobulin superfamily members (i.e. a family including adhesion molecules and cytokine receptors). Secondly, the intracellular signaling and trafficking group included the wide terms Response to zinc and Lysosome, but in addition terms reflecting differences in signaling mediated by Toll-like receptor-linked pathways, MAP kinases and ARF/GTP-ase. The differences in intracellular trafficking involved endocytic uptake. Finally, the third group included responses to pH as well as responses to infections (related to Toll like receptor signaling), regulation of inflammation, and regulation of cell shape.

**Figure 1:**
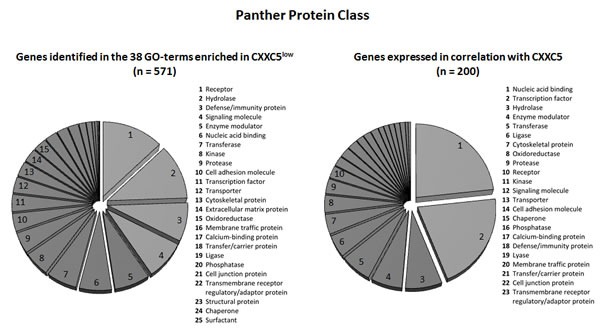
Protein class analysis of the genes showing differential expression for CXXC5^HIGH^ and CXXC5^LOW^ patients in GSEA analysis (left, 571 genes identified) and similarity profiling analysis (right, the 200 genes with the strongest correlation being analyzed) The genes were analyzed according to their functional protein class based on the Panther (http://www.pantherdb.org/) database. The various protein classes were ranked according to the number genes included in each of the classes.

We did an alternative bioinformatical analysis of the 571 genes identified above (Supplementary Figure 2); we then used the Panther database and the genes were analyzed with regard to classification of the encoded proteins (Figure 1 left; total protein class hits 800). The four main classes had the following characteristics (Table 2):

**Table 2:** Comparison of AML cells showing high and low CXXC5 expression - identification and classification of important proteins encoded by the 571 genes identified from the leading edge in the Gene Set Enrichment Analysis (Supplementary Figure 2)

**CLASS 1. RECEPTOR (total protein class hits 68)**	
**1a G-protein coupled receptor (24/68 genes)**	
	The largest subset was chemokine receptors (CCR1/2/7, CXCR1/4, CX3CR1); three adenosine receptors (ADORA3, ADORA2, AADORA2B)
**1b Cytokine receptor (36/68 genes)**	
	LILR and CLEC receptors were also included in this term (see 18b below).
	Interleukin receptors: IL4R, IL8R1, IL10RB, IL18RAP, IL27β
	Others: FAS, TNFRSF1B
**CLASS 2. HYDROLASES (total protein class hits 53)**	
**2a. Phosphatase (9/53 genes)**	
	Including the INPPL1 (phosphatidylinositol 3,4,5-triphosphate 5 phosphatase 2) together with A base subunits (ATP6V0E172), one receptor-type (PTPRJ) and one no receptor (PTPN6) tyrosine-protein phosphatase.
**2b Proteases (35/53 genes)**	
	The complement system: C2, CD45, CFB, CFD, CF1, CR1,
	Cathepsins: CTSA, CTSB, CTSD, CTSG, CTSK, CTSL1, CTSL2, CTSS, CTSZ
	Others: Caspase 1, Matrix metalloprotease 9 and 25.
**CLASS 3. DEFENSE/IMMUNITY PROTEINS (total protein class hits 53)**	
**3a Immunoglobulin receptor superfamily (25/53 genes)**	
	HLA molecules: HLA-A, HLA-B, HLA-C, HLA-E, HLA-F
	C-type lectin receptors (CLEC1A, CLEC5A, CLEC7A), Leukocyte Immunoglobulin Like Receptors (LILRA1-3, LILRB2/3/5).
	Others: CD1D, CD4, Face-receptors (FCGR1A, FCGR1B
**CLASS 4. SIGNALING MOLECULE (Total protein class hits 48)**	
**18a Cytokine (23/48 genes), the main subsets being:**	
	Chemokines: CCL2/3/5/16/20/23, CXCL16
	Receptor for G-CSF
	The Interferon system: IFNA4, IFNA16, IFNB1
	The Interleukin system: (i) IL6 and its downstream targets IL6ST and SOCS3; IL1RA, IL17F, IL27RA
	Lymphotoxin A and TNF
**18b Membrane-bound signaling molecules (17/48 genes), including:**	
	NOTCH2 (LILR and CLEC, see 1b above)

*Defense/immunity proteins.* The majority of genes with different expression were C-type lectin-like receptors (CLEC), HLA molecules (especially class I molecules), and leukocyte immunoglobulin-like receptors (LILR). CLECs and HLA-class I molecules seem to have functional interactions [19] and HLA-molecules are ligands for LILR [20].

*Hydrolases.* The different expression of phosphatidylinositol 3,4,5-triphosphate 5 phosphatase 2 was the only phosphatase suggesting a difference in specific signaling pathways. The most important subset in this class was the hydrolases; differential expression was then seen for several genes encoding members of the complement system, cathepsins that are important for lysosomal functions [21, 22], and the Matrix Metalloprotease 9 that causes proteolytic cleavage of chemokines (e.g. CXCL8, CXCL12) and thereby may be involved in leukemogenesis [23].

*Receptors.* The functions of several cytokine receptors differed, including both the chemokine and the interleukin system as wells as Fas and the G-CSF and TNF receptors. We also observed altered expression of adenosine receptors that may have a role in regulation of apoptosis in human AML [24, 25].

*Signaling molecules.* The expression of cytokines is also altered, including members of both the chemokine, interferon and interleukin systems (especially IL6 signaling).

Thus, the major differences between CXXC5^HIGH^ and CXXC5^LOW^ AML include the following functional networks (Table 2): (i) HLA-class I-CLEC-LILR; (i) the chemokine-chemokine receptor system (including MMP9); (iii) the interleukin system; (iv) the complement system; and (v) the lysosomal function and thereby possibly also regulation of autophagy and cell viability [21, 22].

### CXXC5 expression is correlated with the expression of several hematopoietic transcription factors that are associated with adverse prognosis

Similarity profiling was used to further analyze the global gene expression data and we identified the 200 genes whose expression showed the strongest correlation with CXXC5 expression; those genes with a known function according to the PubMed and Gene databases are listed in Supplementary Table 2. A large subset of these genes is important for gene transcription, the largest group being zinc finger proteins and their interacting proteins. Another large subset was genes important for intracellular signaling including cell surface molecules as well as intracellular mediators. Alternatively, we also investigated the functional importance of the proteins encoded by the 200 genes showing the strongest correlation with CXXC5 expression in the similarity profiling analysis. When this analysis was based on the Panther database we confirmed that a majority of the genes encodes proteins that are important for transcriptional regulation (Figure 1, right). The importance of transcriptional regulation was further confirmed by an alternative analysis based on the Reactome database, and this last analysis in addition suggested that the function of SMAD3 and SMAD4 is altered. The SMAD-signaling pathway operates downstream of the transforming growth factor-β (TGF-β) superfamily of ligands; it seems to be important for regulation of normal and possibly also leukemic hematopoietic stem cell functions including regulation of proliferation, differentiation and apoptosis [26-32]. Finally, some of the terms from the Gene Set Enrichment Analysis were as expected also detected at a lower frequency in the similarity analysis (see Figure 1, right part).

**Figure 2:**
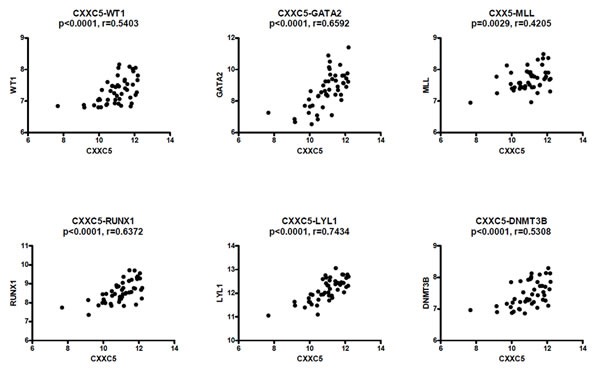
CXXC5/RINF expression is correlated with the expression of several transcriptional regulators in primary human AML cells Global gene expression analyses were performed for 48 consecutive/unselected AML patients, and we then compared expression of CXXC5 with the expression of WT1, GATA2, MLL, RUNX1, LYL1 and DNMT3B.

A subset of AML patients with adverse prognosis shows high expression of certain hematopoietic stem and progenitor cell-associated transcription factors [33-35], especially the heptade SCL, LYL1, LMO2, GATA2, RUNX1, FLI1 and ERG [34]. With regard to this heptade we observed strong correlations between CXXC5 expression and expression of the four genes LYL1 (p<0.0001, r=0.7434), GATA2 (p<0.0001, r=0.6592), RUNX1 (p<0.0001, r=0.6372) and ERG (p=0.0212, r=0.3318); the correlations did not reach significance for the two factors SCL (p=0.1076, r=0.2352) and FLI1 (p=0.1866, r=0.3760) (Figure 2); and even though CXXC5 expression did not show any significant correlation with the expression of the last factor LMO2 (p=0.4815, r=0.1041) it showed a strong correlation with expression of LYL1 that is necessary for recruitment of LMO2 to DNA [36, 37]. Furthermore, CXXC5 expression also showed significant correlations with the expression of several other transcriptional regulators, including WT1 (p<0.0001, r=0.5403), DNMT3B (p<0.0001, r=0.5308), MLL (p=0.0029, r=0.4205), SPI1 (p=0.0001, r=0.5222), MYB (p=0.0004, r=0.4930) and GFI1B (p=0.0110, r=0.3641).

**Figure 3:**
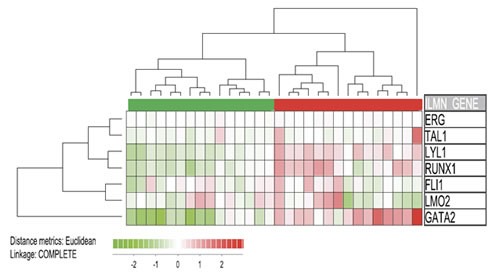
Expression of CXXC5 versus the expression of the transcription factor heptade SCL, LYL1, LMO2, GATA2, RUNX1, FLI1 and ERG The expression of these transcription factors is associated with adverse prognosis [34]. We compared the 15 patients with the highest and the 15 patients with the lowest CXXC5 expression based on our global gene expression profiling for 48 unselected AML patients. We did a hierarchical clustering analysis; based on this transcription factor signature the clustering analysis identified to major subsets corresponding to the CXXC5^HIGH^ and CXXC5^LOW^ patient subsets. Thus, CXXC5 expression is not only associated with the expression of single transcription factors but also with the overall heptade signature.

We finally investigated the association between CXXC5 expression and the overall expression profile of the transcription factor heptade. We then analyzed the expression of all 7 transcription factors for the 15 AML patients with the highest and the 15 patients with the lowest CXXC5 expression (Figure 3), and we did a clustering analysis of these 30 patients based on the expression profile of the 7 factors. The overall heptade signature separated the patients into two main subsets corresponding to the CXXC5^HIGH^ and CXXC5^LOW^ subsets. Thus, clustering analysis shows that CXXC5/RINF expression shows a strong correlation with several other transcription factors involved in hematopoiesis, including several members of the transcription factor heptade (GATA2, RUNX, ERG, the LM02 recruitment factor LYL1) that is associated with adverse prognosis in AML [35].

### High CXXC5 mRNA expression is associated with a stem cell signature that has an adverse prognostic impact

**Figure 4:**
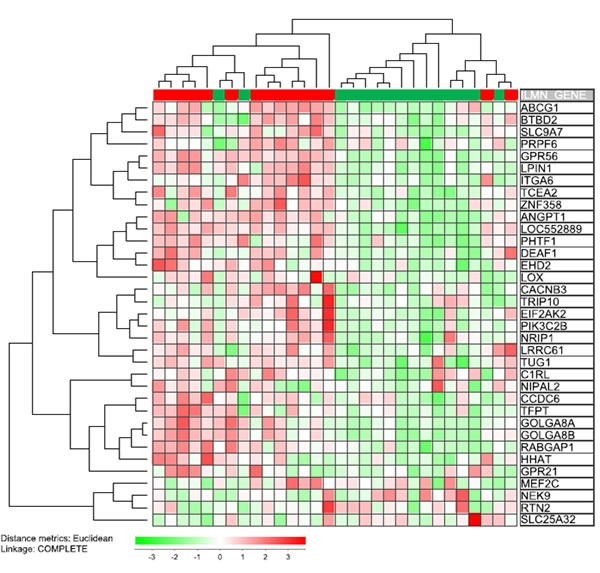
Expression of CXXC5 versus the expression of a stem cell signature associated with adverse prognosis in AML patients receiving intensive chemotherapy [33] The previous study by Eppert et al identified 35 genes that were expressed by leukemic stem cells and associated with an adverse prognosis. We compared the 15 patients with the highest and the 15 patients with the lowest CXXC5 expression in our global gene expression profiling for 48 unselected AML patients. We did a hierarchical clustering analysis. Based on this analysis of the stem cell signature we identified two main patient subset; one subset included the majority of AML patients showing high CXXC5 expression in their leukemic cells (13 out of 15 patients; left main cluster) whereas the other main subset included the majority of patients with low CXXC5 expression (also 13 out 15 patients). Thus, CXXC5 expression is not only associated with the expression of single stem cell associated genes but also with the overall leukemic stem cell signature. All genes included in this analysis (see right part of figure) showed a statistically significant correlation with CXXC5 expression (p<0.05) except for the 9 genes GPR21, LOX, MEF2C, NEK9, NIPAL2, PRPF6, RTN2, SLC25A32, TRIP10.

Eppert et al. [33] identified gene expression signatures for normal hematopoietic and AML stem cells, and three gene subsets were then identified: (i) genes showing differential expression in leukemic stem cells (LSC-related); (ii) genes showing differential expression in normal hematopoietic progenitor/stem cell (HSC-related) with a subset of these genes also being highly expressed in AML stem cells; and (iii) a subset of genes driving the expression of HSC-related genes in AML stem cell. Thereafter they compared the AML cell expression of these genes for patients with good and adverse prognosis, and based on this comparison they identified 35 genes associated with adverse prognosis. Eight of these genes were among the 650 highest ranked genes in the similarity profile analysis and thus showed very strong correlation with CXXC5 expression (GPR56, ANGPT1, SLC9A7, GOLGA8, TUG1, LOC552889, PIK3C2B, RABGAP1; p<0.0001 for all), and CXXC5 expression showed weaker but still significant correlations (p<0.05) with 18 additional genes (Figure 4). We compared the expression of these 35 genes for our 15 patients with the highest and the 15 patients with the lowest CXXC5 expression (Figure 4). Based on this clustering analysis we identified two main patient subsets including a majority (13 out of 15) CXXC5^HIGH^ and CXXC5^LOW^ patients, respectively. Thus, high CXXC5 expression is also associated with this stem cell signature that has an adverse prognostic impact.

### CXXC5^HIGH^and CXXC5^LOW^ AML cells show only minor differences in their cytokine secretion

Our GSEA analysis suggested that regulation of cytokine release (especially IL6 and TNFα) differed between CXXC5^HIGH^ and CXXC5^LOW^ patients, and several of the membrane receptors or intracellular pathways showing differential expression are also important for regulation of cytokine release (see Table 2). For this reason we compared the constitutive cytokine release for the 10 patients in the PCR-analyzed patient cohort with the highest and the 10 patients with the lowest CXXC5 expression in their AML cells. Lenalidomide was also investigated because this drug is used in the treatment of patients with myelodysplastic syndrome (MDS) and the del(5q) abnormality that leads to loss of one CXXC5 gene and low CXXC5 expression (1). Firstly, the constitutive cytokine release did not differ between CXXC5^HIGH^ and CXXC5^LOW^ patients and showed a similar wide variation for both groups without any statistically significant differences (Supplementary Table 3). Secondly, we also did unsupervised hierarchical clustering analyses; the two groups then differed in their constitutive cytokine release clustering and the most striking difference was a very close chemokine clustering (especially CCL2-4 and IL-8/CXCL8) detected only for CXXC5^LOW^ patients (Supplementary Figure 3). The variations between patients could not be explained by differences in spontaneous apoptosis/viability during culture (data not shown). Thirdly, we compared the effect of lenalidomide on the release of individual cytokines (Supplementary Table 4); for the CXXC5^LOW^ group lenalidomide caused a significant reduction of IL1β, TNFα and GM-CSF, whereas for the CXXC5^HIGH^ group a decrease was seen for IL1β, TNFα, IL6 and IL1RA. The percentage reduction of the cytokine levels differed significantly between the two groups only for IL6 (48.1% reduction for CXXC5^HIGH^ versus 78.7% for CXXC5^LOW^ patients, Mann Whitney U-test, p=0.0464). Our gene expression studies suggested differences in IL6 and TNFα release (Table 2), and this was thus supported by our protein studies. However, we conclude that the differences in cytokine clustering and the lenalidomide-induced alterations should be regarded as minor differences in cytokine release; the main difference between individual patients being the wide variation in cytokine release (more than 10^4^-fold for several cytokines, see Supplementary Table 3) that is independent of CXXC5 expression.

### CXXC5^HIGH^ and CXXC5^LOW^ AML cells differ in their intracellular protein phosphorylation profile, including phosphorylation of several transcriptional regulators

Our global gene expression studies suggest that patients with high and low CXXC5 expression differ in their intracellular signaling. We therefore examined the phosphorylation status of 19 intracellular mediators in primary human AML cells derived from 42 unselected patients in our global gene expression patient cohort. The intracellular mediators and the exogenous cytokines added during incubation were selected based on previous observations. Firstly, the intracellular phosphoprotein networks investigated (pathways or single mediators integrating signaling through several pathways) are important for downstream signaling from growth factor receptors commonly expressed by primary human AML cells [12, 13]. Secondly, ligation of these growth factor receptors by exogenous cytokines not only initiates alterations in protein phosphorylation but also increases [38-40] primary AML cell proliferation and/or modulates cytokine-dependent proliferation [41] for a majority of patients when tested in standardized *in vitro* models.

**Figure 5:**
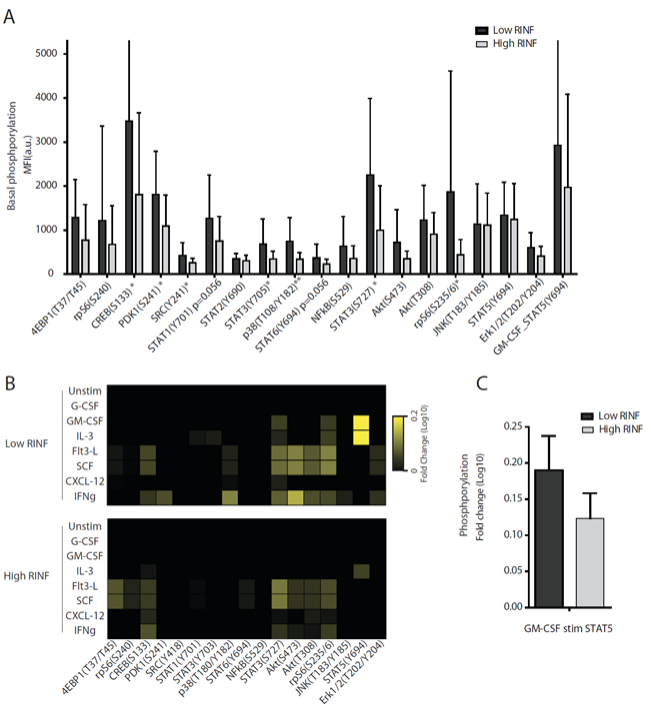
The intracellular phosphorylation status and phospho-responsiveness of primary human AML cells derived from patients with high and low constitutive mRNA CXXC5/RINF expression A total of 42 patients were included in these studies, and the phosphoprotein status was examined by flow cytometry. (A) The basic phosphorylation status was compared for the 21 patients with the highest and the 21 patients with the lowest CXXC5 expression. The results are presented as the mean and standard deviation for the MFI values. Significant differences between the two groups are indicated in the figure (* p<0.05, ** p<0.01). (B) We then compared the phosphorylation responsiveness for the 7 patients with lowest CXXC5/RINF expression and the 10 patients with the highest CXXC5/RINF mRNA levels. The leukemic cells were cultured with 7 exogenous cytokines (see left margin), and median fold alteration (see right margin) for each cytokine/mediator is presented for each of the two groups. (C) The fold change of STAT5 phosphorylation was also compared for the 21 CXXC5^LOW^ and CXXC5^HIGH^ patients; these results are presented as the fold change.

The patients showed an expected wide variation of CXXC5 expression in their AML cells. As explained above all the 19 intracellular mediators can be constitutively phosphorylated and/or become phosphorylated in response to cytokine exposure of primary human AML cells, but our high-throughput flow-cytometry technique required an incubation prior to analysis in culture medium containing insulin and transferrin [12, 13, 42]. This medium was used because it is suitable for extended *in vitro* culture of primary human AML cells [43, 44]. Our basal phosphorylation status thus refers to the phosphoprotein profile under the influence of these two mediators but without additional hematopoietic growth factors. The comparison of the basal phosphorylation for the 21 patients with the highest and the 21 patients with the lowest CXXC5 expression showed several differences in intracellular signaling and transcriptional regulation with increased phosphorylation of CREB (S133), PDK1 (S241), Src (Y241), STAT3 (Y705), STAT3 (S727), p38 (Y182) and rpS6 (S235/6) for the CXXC5^LOW^ patients (Figure 5A). Several of these mediators are involved in PI3K-Akt-mTOR mediated signaling, including PDK1 [45], Src [46], rpS [47], and the transcription factor CREB that is a downstream target of the pathway [48]. STAT3 can also can be activated by Src in addition to g-protein receptors [49]. Finally, p38 is activated by several cytokines and is an important regulator of hematopoiesis through transcription factor activation [50].

We also did a similar comparison based on the PCR analysis of the qPCR cohort; we then compared the phosphoprotein profiles for the 10 patients with the highest and the 7 patients with the lowest CXXC5 expression. We detected increased phosphorylation also of several additional intracellular mediators for CXXC5^LOW^ patients (p<0.05 for all). Firstly, STAT1 (Y701) phosphorylation was increased; this mediator has an overlapping repertoire with STAT3 [49] and is a downstream target of Akt [46]. Secondly, STAT6 (Y694) phosphorylation was increased. STAT6 is activated by IL4/IL13 that reduce the constitutive release of several cytokines by primary human AML cells [51, 52]. STAT6 is also a regulator of normal hematopoiesis [53] and several genetic loci encoding chemokines and adhesion molecules contain STAT6-binding motifs [54]. Finally, ERK1/2 phosphorylation (T202/Y204) was altered and CREB is one of its downstream targets [55].

We finally compared the alterations in phosphorylation status when cells were incubated with 7 exogenous cytokines. The overall phosphoresponse differed between CXXC5^HIGH^ and CXXC5^LOW^ patients from the PCR cohort, the most striking difference being that only CXXC5^LOW^ patients showed an increased phosphorylation of STAT3(S727), rpS6(S235/6) and STAT5(Y694) in response to GM-CSF (Figure 5B). Only minor differences were detected for other mediators in response to IL3, Flt3-L, SCF, CXCL12 and IFNγ. Similar GM-CSF-induced alterations were detected when comparing the 21 CXXC5^HIGH^ and the 21 CXXC5^LOW^ patients in the microarray patient cohort (Figure 5C).

### Inhibition of CXXC5 expression by pharmacological intervention

**Figure 6:**
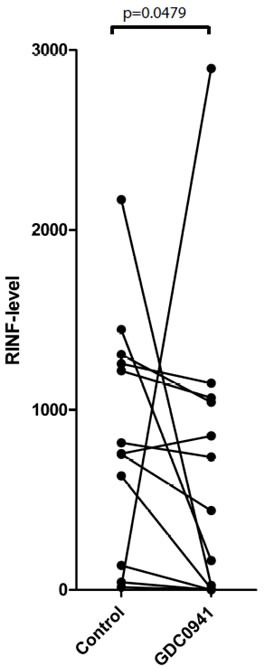
The effect of pharmacological inhibition on CXXC5/RINF mRNA expression levels Primary human AML cells were cultured with GDC0941 for 5 hours before CXXC5/RINF mRNA levels were compared for drug-free control cultures and drug-containing cultures (Wilcoxon's test for paired samples, p=0.0479).

We investigated pharmacological effects on CXXC5 expression for primary human AML cells after 5 hours of *in vitro* exposure. CXXC5 expression showed a difference of only borderline significance for the pan-PI3K inhibitor GDC0941 (Figure 6, p=0.0479). We observed divergent effects without statistically significant alterations for the PKC agonist PEP005, lenalidomide, the proteasomal inhibitor bortezomib, the mTOR inhibitor rapamycin, the HSP90 inhibitor 17-DMAG, the HSP70 inhibitor VER-155008 and the protein kinase inhibitors PD98059, SB202190 and SP600125 (data not shown). These data suggest that CXXC5 expression integrates signaling through various pathways, and the contribution of each pathway differs between patients.

### Effects of CXXC5 knockdown on gene expression in human AML cells

CXXC5 was knocked down by lentiviral vectors in three different AML cell lines; one vector with pyromycine selection was used for MV4-11 (759 genes upregulated and 744 genes downregulated at least 1.5 fold) and K562 cells (870 genes upregulated and 758 genes downregulated at least 1.5 fold) and another vector based on GFP sorting was used for knockdown in K562 (356 genes upregulated and 457genes downregulated at least 1.5 fold) and UT7 cells (1291 genes upregulated and 827 genes downregulated at least 1.5 fold). CXX5 knockdown was documented both at the mRNA and protein level for all experiments. These genes were compared with the 50 genes showing highest mRNA expression in the CXXC5^HIGH^ and CXXC5^LOW^ patient subset (see Supplementary Tables 1 and 2).

Global gene expression profiles were compared for cells transfected with knock-down vectors and emty control vectors. Four genes were identified according to the following criteria: (i) being among the 50 genes with highest expression in CXXC5^HIGH/LOW^ patients (Supplementary Tables 5 and 6); and showing either (iia) >2-fold alteration after CXXC5 knockdown for one cell line or (iib) >1.5-fold alteration for two different cell lines. All four genes showed high levels for CXXC5^HIGH^ patients increased levels after CXXC5 knockdown, and their relevance for carcinogenesis/leukemogenesis is described in detail in Suppl. Table 7 and summarized below.

*Increased levels of the potential tumor suppressor gene TSC22D1.* This gene (TSC22 domain family, member 1) shows increased levels after CXXC5 knockdown and encodes a transcriptional regulator [56] that is a potential tumor suppressor in human AML [57]; it can also contribute to induction of apoptosis in human gastric and breast cancer cells and increase cancer cell sensitivity to anticancer drugs [56] and radiation [58].

*Increased levels of the cell cycle regulator SEPP1.* The encoded mediator (Selenoprotein P.plasma.1) is a secreted glycoprotein containing extracellular selenium [59]. Selenium and its metabolites are involved in regulation of apoptosis and proliferation/cell cycle progression [60].

*Increased expression of the receptor for the Stem cell factor (KIT) and for Angiopoietin 1 (ANGPT1). KIT* shows increased levels and encodes CD117 that is expressed by primary human AML cells for most patients but its expression has no prognostic impact for AML patients receiving intensive chemotherapy [61]. *ANGPT1* (Angiopoietin 1) is a Tie2 agonist that shows increased expression in CXXC5^HIGH^ cells. Angiopoietin 1 is a regulator of angiogenesis without any prognostic impact by itself in human AML, whereas extracellular levels (serum, bone marrow plasma) of the potential Tie2 antagonist Angiopoietin 2 has an adverse prognostic impact in human AML [62-64]. Finally, ANGP1 is also a member of the leukemic stem cell gene expression signature that has been associated with adverse prognosis in human AML [33]. The extracellular release of Angiopoietin 1 showed a wide variation during *in vitro* culture (median 37 pg/ml, range <0.5 pg/ml to 2135 pg/ml) and a correlation was seen between the level of Angiopoietin 1 mRNA expression and the level of protein release (p=0.001).

The expression of the transcriptional regulators showing correlated mRNA expression with CXXC5 was not altered by the knockdown (see above).

### CXXC5 expression increases during progression of chronic myeloid leukemia to blast phase

**Figure 7:**
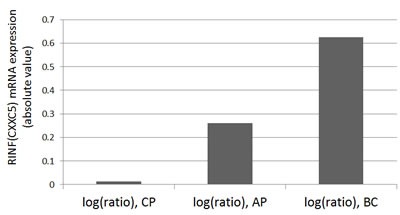
CXXC5/RINF is increased during the progression of chronic myeloid leukemia from chronic phase to blast crisis The microarray data have been performed by Radich JP et al. [65]. The CXXC5/RINF gene expression data were extracted from a gene list (supporting table 4 or 10423Table4.xls) available online at the PNAS website (http://www.pnas.org/content/103/8/2794/suppl/DC1). CP: chronic phase (n=42), AP: accelerated phase by blast count criteria (n=9) or by the occurrence of additional clonal cytogenetic changes (n=8), BC: blast crisis (n=28).

We previously described that CXXC5 is expressed in AML as well as acute lymphoblastic leukemia cells [4]. In our present study we compared CXXC5 expression in chronic myeloid leukemia cells; we then used previously published global gene expression data [65]. CXXC5 expression was low for CML bone marrow cells in chronic phase, the levels increased during accelerated phase and were highest for patients in blast phase (Figure 7). Thus, high/increasing CXXC5 expression is associated with more aggressive disease not only in AML but also in CML.

## DISCUSSION

The CXXC5 gene seems important during both normal and leukemic hematopoiesis [2]. High expression in the leukemic cells is an independent adverse prognostic factor in AML [5] and possibly associated with adverse prognosis also in other myeloproliferative diseases, e.g. CML [66].

CXXC5 encodes a transcriptional regulator [3]. Our present studies suggest that this protein is a part of a larger transcription-regulatory network that differs between CXXC5^LOW^ and CXXC5^HIGH^ patients and includes several stem cell associated transcription factors as well as a heptade of transcription factors associated with an adverse prognosis in human AML [34]. Our bioinformatical analyses demonstrated that CXXC5 expression showed a strong association both to (i) the overall expression profiles of the transcription factor heptade associated with adverse prognosis (Figure 3); and (ii) the overall signature of 35 stem cell-associated genes used to identify AML patients with high-risk disease (Figure 4). CXXC5 expression levels also showed significant correlation with several genes included both in the transcription factor heptade and in the stem cell gene expression profile. Thus, the association between high CXXC5 expression and adverse prognosis is seen in a biological context of a specific transcription-regulatory network and an AML stem cell-associated expression signature. The hypothesis that CXXC5 is a part of a larger transcription-regulatory network is also supported by our similarity gene expression analysis and the corresponding protein function analysis (Figure 1).

Our comparison of gene expression profiles in contrasting CXXC5^HIGH^ and CXXC5^LOW^ patients identified 571 genes with different expression (Supplementary Figure 2). The analysis of corresponding protein functions suggests that these two contrasting groups show major differences with regard to communication between cells, i.e. expression of various cell surface molecules (including cytokine receptors) and release of soluble mediators (Figure 1, Table 2). However, there was also an overlap between protein functions identified through the CXXC5 similarity expression analysis and the GSEA analysis of contrasting patient subsets (Figure 1, left part).

We compared the constitutive cytokine release for primary human AML cells with high and low CXXC5 expression but could not detect any difference when comparing single cytokine levels (Supplementary Table 3) but only with regard to cytokine clustering (Supplementary Figure 3). The effect of lenalidomide on cytokine release also differed between the subsets, and these effects could not be explained by a nonspecific effect secondary to altered viability because a similar effect on all cytokines would then be expected. However, the wide variation in constitutive release is definitely the most important difference between AML patients and this variation is similar for CXXC5^HIGH^ and CXXC5^LOW^ patients; the differences in cytokine clustering and lenalidomide effects should only be regarded as minor differences.

We compared the intracellular phosphoprotein status for CXXC5^HIGH^ and CXXC5^LOW^ AML cells. Our high-throughput technique requires an incubation step for antibody binding [14]; we then used a medium suitable for immature hematopoietic cells and containing insulin and transferrin but no hematopoietic growth factors [43, 44] and we refer to this as basal phosphorylation status. In addition we compared growth factor-induced alterations of protein phosphorylation. The low-risk CXXC5^LOW^ patients showed increased basal phosphorylation of several mediators. Additional differences were induced by exogenous growth factors, especially GM-CSF/IL3-induced increase of STAT5 phosphorylation. Thus, variation in CXXC5 expression is associated with differences in intracellular signaling targeting downstream transcription factors.

Our studies suggest that signaling though PI3K-Akt is important for CXXC5 expression, and several of the mediators showing different phosphorylation for CXXC5^HIGH^ and CXXC5^LOW^ patients are downstream targets to PI3K-Akt-mTOR (see above). Some of the genes identified in the CXXC5 similarity profiling are also important for this pathway (Supplementary Table 2A). All these observations suggest that the pathway is important for regulation of CXXC5 expression. Finally, pharmacological inhibition of other intracellular pathways had divergent effects, these observations then being consistent with the hypothesis that regulation of CXXC5 mRNA expression differs between patients and depends on several/various intracellular signaling pathways.

Previous studies suggest that high constitutive signaling through the PI3K-Akt-mTOR pathway [67, 68] and activation of its downstream target STAT3 [69] is associated with adverse prognosis in human AML. However, a recent study suggested that AML patients are heterogeneous and signaling through this pathway can cause growth inhibition as well as growth enhancement in primary AML cells [70]. STAT3 shows an extensive and cell type-dependent variation in its preference to potential DNA binding sites [71, 72], and a similar variation between biologically heterogeneous AML patients may explain the different biological effects of PI3K-Akt-mTOR-STAT3 signaling between AML patients [73]. Furthermore, the possible prognostic impact of STAT3 activation in human AML is also controversial. Benekli et al. [69] described an association between high constitutive STAT3 activation (i.e. high Y705 phosphorylation) and adverse prognosis, whereas a recent study described improved survival for patients showing high STAT3 Y705 and S727 phosphorylation in their AML cells in response to cytokine stimulation [74]. This last observation is consistent with our present observation of an association between high STAT3 phosphorylation for lowrisk CXXC5^LOW^ patients. One possible hypothesis for these apparently conflicting observations could be that the prognostic impact of STAT3 in human AML is not (only) mediated by its phosphorylated form, but by the unphosphorylated form and its interaction with NFκB signaling [72]. This hypothesis is also consistent with the previously described associations between phosphorylated forms of STAT3 and antiproliferative/proapoptotic effects in human cancer cells [75] and stem cells [76], during murine carcinogenesis [77] and in STAT3 knock-down models [78].

STAT5 phosphorylation/activation can also mediate proapoptotic effects through activation of growth inhibitory and proapoptotic genes depending on its biological context [79], and this may also explain the association between the CXXC5^LOW^ phenotype and increased STAT5 phosphorylation after GM-CSF/IL3 exposure. Finally, increased CREB phosphorylation was observed for CXXC5^LOW^ patients, but this may be less important because animal models suggest that CREB only contributes to the AML phenotype but is not sufficient for leukemic transformation [48]. Thus, the increased phosphorylation of these intracellular mediators for lowrisk CXXC5^LOW^ patients may contribute to the good prognosis for these patients due to the biological context, i.e. crosstalk between pathways or interactions with other transcriptional regulators [80].

Based on the knockdown experiments and analysis of global gene expression profiles (Supplementary Tables 5 and 6) we identified four genes that both were altered by CXXC5 knockdown and whose expression showed a strong correlation with CXXC5 expression in primary AML cells. These genes were increased by CXXC5 knockdown; this can be explained by an inhibitory effect of CXXC5/RINF on their expression and stimulatory effect(s) by other transcriptional regulators that show strong correlation with CXXC5 expression (see above). Those genes showing high expression in CXXC5^HIGH^ primary human AML cells and being significantly altered (i.e. increased) after CXXC5 knockdown included one potential tumor suppressor (TSC22), the cytokine Angiopoietin 1, a selenium transport protein and the hematopoietic growth factor receptor KIT. The three first may be important for regulation of apoptosis or cell cycle progression in human malignant cells, whereas the KIT receptor mediates growth-enhancing effects in human AML cells although it does not have any impact on chemosensitivity/prognosis (Supplementary Table 7). Thus, these knockdown experiments further support a role of CXXC5/RINF in AML chemosensitivity.

To summarize, CXXC5 expression shows a wide variation in primary human AML cells and high levels are associated with an adverse prognosis. Our present results suggest that CXXC5 integrates several intracellular signaling events and thereby have effects of several steps in regulation of leukemogenesis or chemosensitivity in human AML.

## SUPPLEMENTARY FIGURES AND TABLES


