# Supplementary material for: Expression of the potential therapeutic target CXXC5 in primary acute myeloid leukemia cells - high expression is associated with adverse prognosis as well as altered intracellular signaling and transcriptional regulation
Source: Oncotarget. 2014 Dec 26;6(5):2794–811. doi: 10.18632/oncotarget.3056 (PMC4413618; doi:10.18632/oncotarget.3056)
Supplement: Supplementary file 1 [file oncotarget-06-2794-s001.pdf]

Expression of the potential therapeutic target CXXC5 in primary acute myeloid leukemia cells - High expression is associated with adverse prognosis as well as altered intracellular signaling and transcriptional regulation

Supplementary Material

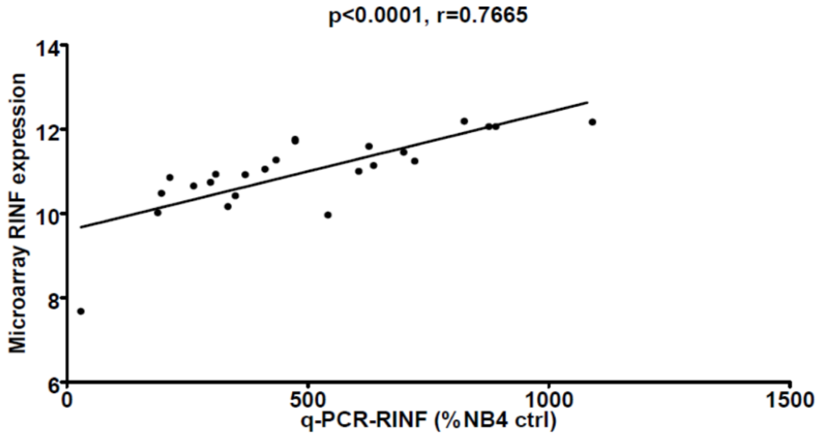

Supplementary Figure 1:

CXXC5 mRNA levels were determined for primary human AML cells derived from 24 unselected patients. A statistically significant correlation was detected between levels determined by PCR and microarray analyses.

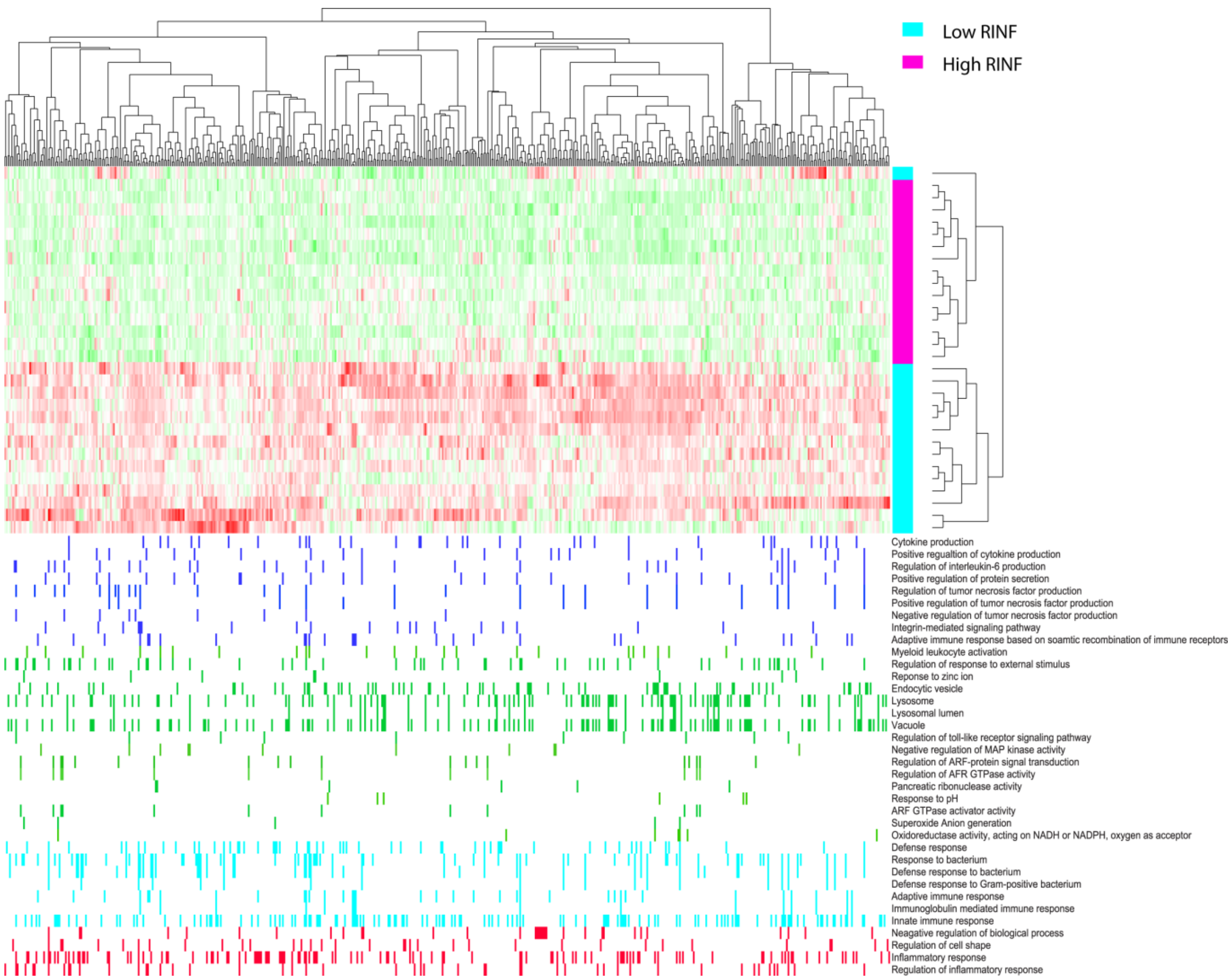

Supplementary Figure 2:

Global gene expression analysis of primary human AML cells was performed for 48 consecutive/unselected patients. We did a Gene Set Enrichment Analysis (GSEA) as a comparison between the 15 patients with the highest and the 15 patients with the lowest CXXC5 expression among these 48 patients. This

analysis identified 38 GO-terms with a false discovery rate (RDR) <1.0. All GO-terms were enriched in the CXXC5^{LOW} group (red colour); and we identified 571 genes that belonged to the leading edge for at least one of these 36 terms. When these 571 genes were used in a hierarchical clustering analysis we identified two main subsets corresponding to the original CXXC5^{LOW} and CXXC5^{HIGH} patient subsets (see left part of the figure). The 38 identified GO-annotations are given in the right top of the figure; and it is also indicated which genes that belonged to each of this terms.

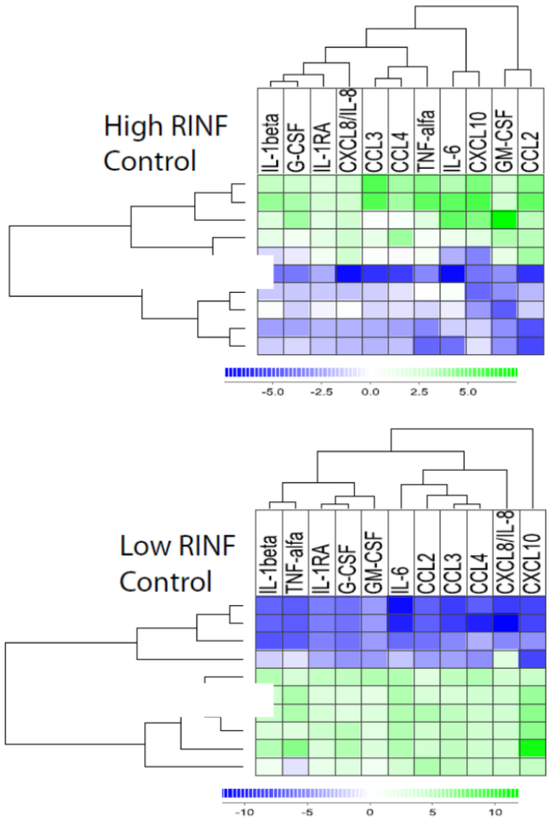

Supplementary Figure 3:

Constitutive cytokine release profiles for primary human AML cells with high and low CXXCS/RINF mRNA expression. Primary human AML cells derived from 10 patients with high and 10 patients with low RINF expression were cultured for 24 hours before cytokine levels {Ill, lllRA, IL6, IL8, TNFa, G-CSF, GM-CSF, CCL2, CCL3, CCL4 or CXCLlO) in the culture supernatants were determined. Hierarchical cluster analyses were used to analyze the constitutive cytokine release profiles for the two groups. The cytokine clustering differed between the two patients subsets.

Supplementary Table 1:
Global gene expression profiles for patients with high and low CXXC5 expression. We analyzed the global gene expression profiles for 48 consecutive/unselected AML patients and did a Gene Set Enrichment Analysis where we compared the 15 patients with highest and the 15 patients with the lowest CXXC5 expression. The GO-terms with a FDR<1 are listed below (bold). The comments given in parentheses for some of the terms are based on information from the PubMed and Gene databases.

EXTRACELLULAR COMMUNICATION

Cytokine production
Positive regulation of cytokine production
Regulation of Interleukin 6 production (IL6 expression can be induced in various cells by IL1 and TNF via NFκB)
Positive regulation of protein secretion
Regulation of tumor necrosis factor production (TNF is an regulates immune responses as well as leukemic hematopoiesis
Positive regulation of tumor necrosis production
Negative regulation of tumor necrosis factor production
Integrin-mediated signaling pathway (these transmembrane receptors mediate attachment between cells and their surroundings, e.g. other cells or extracellular matrix)
Adaptive immune response based on somatic recombination of immune receptors built from immunoglobulin superfamily (this family includes molecules involved in initiation of antigen-specific immune responses but in addition several molecules involved in intercellular communication and intracellular signaling, i.e. the adhesion molecule ICAM-1, cytokine receptors (IL6, PDGF, CD115, CD117), receptor protein tyrosine phosphatases and the MMP induced CD147

INTRACELLULAR SIGNALING

Myeloid leukocyte activation
Regulation of response to external stimulus
Response to zinc ion (zinc is important both for extracellular communication and intracellular signaling).
Endocytic vesicle (endocytic vesicles are an intracellular compartment being a part of the endocytic membrane transport pathway from the plasma membrane to the lysosome)
Lysosome
Lysosomal lumen
Vacuole
Regulation of Toll-like receptor signaling pathway (downstream targets including PI3K-Akt, MAP kinases and NFκB)
Negative regulation of MAP kinase activity (Mitogen-activated protein (MAP) kinases are serine/threonine-specific protein kinases involved in cellular responses to several stimuli including proinflammatory cytokines, and they regulate proliferation, gene expression, differentiation and cell survival/apoptosis)
Regulation of ARF protein signal transduction (ARF regulates transcription and acts as a tumor suppressor through interactions with MDM2/ p53)
ARF GTP-ase activator activity
Regulation of ARF GTP-ase activity
Pancreatic ribonuclease activity (proangiogenic angiogenin belongs to this superfamily)

OTHERS

Defense response, Response to bacterium, Defense response to bacterium, Defense response to Gram-positive bacterium, Inflammatory response, Regulation of inflammatory response, Adaptive immune response, Immunoglobulin-mediated immune response, Innate Immune response.
Negative regulation of biological process, Regulation of cell shape
Response to pH, Superoxyd anion generation, Oxidoreductase activity, acting on NAD or NADP,oxygen as acceptor

Supplementary Table 2A:
Similarity profiling analysis - identification of genes showing a similar expression profile as CXXC5. Based on the global gene expression profiles for 48 consecutive patients we identified the 200 genes with the most similar expression, the table shows the classification of the 145 genes with a known function. The classification is based on information from the PubMed and Gene databases.

Classification of the genes	Individual genes whose expression is correlated with CXXC5 expression	Number of genes
**Regulators of transcription**		**66**
Zinc finger proteins and proteins interacting with zinc finger protein	ZNF792 (126375); ZCCHC3 (85364); ZNF313 (55905); ZNF395 (55893); ZSCAN18 (65982); ZNF320 (162967); ZBTB42 (1001128927), ZNF428 (126299); ZNF254 (9534); VEZF1 (7716); ZNF22 (55906); ZC4H2 (55906); TEX10 (54881); ZFP1 (162239); ZNF146 (7705); ZNF17 (7565); ZNF439 (90594); ZNHIT6 (54680); ZNF165 (7718); ZMYM4 (9202); ZBTB8A (653121); ZNF644 (84146); ZNF550 (162972); PHF15 (23338); ZNF490 (57474); PLAGL1 (5325).	26
Transcription factor	RFX7 (64864); TGIF2 (60436); DPF2 (5977); SP4 (6671); HHEX (3087); SOX4 (6659); CUTA (51596); ETV6 (2120); TSC22D1 (8848); GATA2 (2624); NONO (4841); ELF1 (1997); RAI1 (10743).	13
Proteins directly interacting with transcription factors or otherwise regulating gene transcription	NAB1 (4664); LYL1 (4066); SET (6418); SIN3A (25942); MAGED2 (10916); SBF1 (6305); PHF16 (9767); ASH2L (9070); PHF14 (9678); SMARCAL1 (50485); SMARCD1 (6602); CNOT2 (4848); KDM5B (10765); RNF144A (9781); KLHDC2 (23588).	15
Proteins showing RNA binding or being involved in RNA metabolism, splicing, transport, translation	KHDRBS1 (10657); DARS (1615); HNRNPA0 (10949); SFRS5 (6430); EDC3 (80153); GAR1 (54433); SFRS6 (6431); DDX1 (1653); QARS (5859); RPL22 (6146); SRBD1 (55133); PWP1 (11137); MPHOSPH10 (10199); RPL3 (6122); FXR1 (8087); TRMT11 (60487).	12
**Intracellular signaling**		**51**
Cell surface membrane molecules	VEZT (55591); C16ORF58 (64755); TSPAN3 (10099); TAPT1 (202018); TMEM57 (55219).	5

G-proteins and their signaling	GUCY1A3 (2982); RRAGA (10670); RAB37 (326624); ANKMY2 (57037); GNA15 (2769); RALA (5898); GPR114 (221188); PDCL (5082); GPSM1 (26086); ARHGAP23 (57636).	10
PI3K-Akt-mTOR signaling	IP6K1 (9807); IP6K2 (51447); WDR48 (57599); MBOAT4 (79143).	4
Intracellular transport/trafficking and protein degradation	C11ORF2 (738); LEPROTL1 (23484); USP11 (8237); ELMO1 (9844); PEX5 (5830); USP38 (84640); TTC3 (7267); NUFIP1 (26747); ARIH2 (10425); ATP8B2 (57198); COG6 (57511).	11
Microtubuli-associated proteins, cytoskeleton	MAPRE2 (10982); APPBP2 (10513).	2
Other proteins involved in intracellular signaling	PTPRCAP (5790); PDE6B (5158); TFPI (7035); ELMO1 (9844); KHDRBS1 (10657); AKR1C3 (8644); TGFBRAP1 (9392); PRKCQ (5588); CASP6 (839); CDK6 (1021); OSBPL9 (114883); ORAI3 (93129); DUSP14 (11072); MAGED1 (9500); KLHL6 (89857); CNOT7 (29883); NLRC3 (197358); DCAF7 (10238); WDR6 (11180).	19
**Mitochondrial proteins**		**10**
	RDH14 (57665); ATP1B1 (481); ALDH5A1 (7915); REPIN1 (29803); ACSS1 (84532); BCKDHB (594); TOMM20 (9804); GPAM (57678); TOMM70A (9868); MRPS31 (10240);	
**Cellular stress responses**		**4**
Antioxidant deficiency, genome stability and DNA repair	SSBP2 (23635); LANCL1 (10314); PRDX6 (9588); KLHL24 (54800).	
**Additional genes with known function**		**17**
	KIFAP3 (22920); OCIAD2 (132299); PARP1 (142); KLHL9 (55958); ASMTL (8623); C1ORF71 (163882); DDHD2 (23259); ACTR8 (93973); KIAA0748 (9840); MBLAC2 (153364); LBA1 (9881); LASS6 (253782); SCAP (22937); METAP1 (23173); TRIM68 (55128); PPAT (5471); CIRBP (1153).	

Supplementary Table 2B.

Global gene expression analyses of patients with high and low expression of CXXC5 - the results from similarity profile analysis. We investigated the global gene expression profile for 48 consecutive/unselected patients. The table gives the 200 genes with the largest similarity to CXXC5.

ILMN_GENE	ENTREZ_GENE_ID
CXXC5	51523
RFX7	64864
CCDC102A	92922
ZNF792	126375
TGIF2	60436
KIAA1147	57189
DPF2	5977
GUCY1A3	2982
LOC552889	552889
ZCCHC3	85364
SSBP2	23635
HS.213061	
SP4	6671
RRAGA	10670
C11ORF2	738
HS.213541	
PTPRCAP	5790
RAB37	326624
HNRNPA0	10949
NAB1	4664
RDH14	57665
PDE6B	5158
LEPROTL1	23484
LOC284422	284422
EPDR1	54749
TFPI	7035
LYL1	4066
C3ORF54	389119
ATP1B1	481
USP11	8237
ANKMY2	57037
LOC729446	729446
ELMO1	9844
CHST12	55501
RNF114	55905
IP6K1	9807
ZNF395	55893
HS.91389	
GNA15	2769
C20ORF11	54994
KHDRBS1	10657
LANCL1	10314
KIFAP3	22920

ALDH5A1	7915
DARS	1615
MAPRE2	10982
PEX5	5830
RALA	5898
OCIAD2	132299
LOC100129484	100129484
PARP1	142
KLHL9	55958
LOC550643	550643
SFRS5	6430
AKR1C3	8644
ZSCAN18	65982
HHEX	3087
EDC3	80153
TGFBRAP1	9392
REPIN1	29803
SET	6418
PRKCQ	5588
GPR114	221188
ZNF320	162967
VEZT	55591
C1ORF165	79656
GAR1	54433
SOX4	6659
PDCL	5082
ZBTB42	100128927
ZNF428	126299
USP38	84640
ZNF254	9534
KIAA0182	23199
ASMTL	8623
C1ORF71	163882
SIN3A	25942
HS.568928	
LOC731366	731366
ACSS1	84532
LOC729102	729102
IP6K2	51447
SFRS6	6431
CUTA	51596
GPSM1	26086
VEZF1	7716
DDHD2	23259
ZNF22	7570
ZC4H2	55906
TTC3	7267
BCKDHB	594
TEX10	54881
APPBP2	10513
DDX1	1653
NUFIP1	26747

ACTR8	93973
ZFP1	162239
TOMM20	9804
HS.133324	
ZNF146	7705
MAGED2	10916
FLJ13197	79667
CASP6	839
SBF1	6305
C13ORF23	80209
CDK6	1021
KIAA0748	9840
QARS	5859
C16ORF58	64755
RPL22	6146
LOC642357	642357
TSPAN3	10099
TAPT1	202018
PHF16	9767
PWP1	11137
MBLAC2	153364
ARIH2	10425
LBA1	9881
ASH2L	9070
OSBPL9	114883
ORAI3	93129
DUSP14	11072
MAGED1	9500
LASS6	253782
ZNF17	7565
REXO4	57109
ETV6	2120
BEND5	79656
SRBD1	55133
PHF14	9678
ATP8B2	57198
TSC22D1	8848
WDR48	57599
EHBP1	23301
MPHOSPH10	10199
MBOAT7	79143
KLHL6	89857
HS.572538	
KIAA1310	55683
HS.355933	
ZNF439	90594
CNOT7	29883
LOC25845	25845
SCAP	22937
SMARCAL1	50485
LOC145783	145783
LOC647346	647346

ZNHIT6	54680
ZNF165	7718
ZMYM4	9202
METAP1	23173
RPL3	6122
C11ORF49	79096
LOC650369	650369
ZBTB8A	653121
PRDX6	9588
TRIM68	55128
GATA2	2624
FXR1	8087
GPAM	57678
NLRC3	197358
TOMM70A	9868
ZNF644	84146
MRPS31	10240
TIGD2	166815
SMARCD1	6602
CNOT2	4848
LOC648980	648980
PPAT	5471
CEP68	23177
HS.405877	
NARG2	79664
KDM5B	10765
DCAF7	10238
RNF144A	9781
RAVER2	55225
LOC388275	388275
TMEM57	55219
WDR6	11180
ZNF550	162972
NUCKS1	64710
CIRBP	1153
LOC643310	643310
KLHL24	54800
NONO	4841
PHF15	23338
NANOS3	342977
ELF1	1997
ARHGAP23	57636
ZNF490	57474
KLHDC2	23588
PLAGL1	5325
RNF144	9781
TRMT11	60487
COG6	57511
MSH6	2956
DHRS11	79154
HNRNPA1L2	144983
RAI1	10743

DMAP1	55929
LOC730246	730246

Supplementary Table 3.
Constitutive cytokine release by primary human AML cells with low and high CXXC5 expression; a comparison of the 10 patients with the highest and the 10 patients with the lowest expression (PCR analysis) among 67 consecutive patients.

**Cytokine**	**Low RINF expression**	**High RINF expression**
IL1β	1329 (<2.0- >9392)	31.7 (2.6- 614.3)
IL1RA	524.7 (<1.8- 2560)	86.3 (15.3- 377.6)
IL6	12855 (<1.4- >19170)	494.4 (3.6- >19170)
IL8	26604 (<1.7- >26604)	15760 (34.8- >26604)
G-CSF	967.9 (<2.0- 6213.5)	16.6 (<2.0- 336.0)
GM-CSF	59.4 (<0.8- 663.0)	8.9 (<0.8- 1224.0)
TNFα	2646.5 (<4.0- >76357)	45.6 (<4.0- 1585)
CCL2	2067 (<1.9- 11281)	956.5 (3.3- 7506)
CCL3	11580.2 (<1.3- >17349)	269.2 (5.7- >17349)
CCL4	23517 (<1.2- >25602)	1511 (41.7- >25602)
CXCL10	7146 (<0.8- >32358)	733.0 (76.7- >32358)

Cells were cultured for 48 hours before cytokine levels were determined in the culture supernatants. We investigated the levels for 10 patients with low (120.6, range 28.7- 213.4) and 10 patients with high (919.6, range 265.6-1476.6) CXXC5 expression analyzed by the PCR assay.

Supplementary Table 4

Effect of lenalidomide on the constitutive cytokine release by primary human AML cells; a comparison of leukemic cells with high and low RINF expression (i.e. the 10 patients with the highest and the 10 patients with the lowest expression among 67 consecutive patients, CXXC5 expression analyzed by PCR).

	**LOW RINF EXPRESSION**			**HIGH RINF EXPRESSION**		
**Cytokine**	**Medium alone**	**Lenalidomide**	**p-value**	**Medium alone**	**Lenalidomide**	**p-value**
Il1β	1329 (<2.0- >9392)	258.9 (<2.0- 3755)	**0.0156**	31.7 (2.6- 614.3)	20.6 (<2.0- 113.5)	**0.002**
Il1RA	524.7 (<1.8- 2560)	471.3 (<1.8- 1727)	0.0781	86.3 (15.3- 377.6)	61.7 (<1.8- 212.0)	**0.0273**
IL6	12855 (<1.4- >19170)	5022 (<1.4- >19170)	0.1250	494.4 (3.6- >19170)	166.5 (2.1- >19170)	**0.0039**
TNFα	2646 (<4.0- >76357)	302.6 (<4.0- 4739)	**0.0156**	45.6 (<4.0- 1585)	17.9 (<4.0- 228.0)	**0.0156**
GM-CSF	59.4 (<0.8- 663.0)	27.2 (<0.8- 173.2)	**0.0313**	8.9 (<0.8- 1224.0)	4.7 (<0.8- 399.6)	0.0625

Cells were cultured for 48 hours in medium alone or medium with lenalidomide 0.5µM before cytokine levels were determined in the culture supernatants. We investigated the levels for 10 patients with low (120.6, range 28.7-213.4) and 10 patients with high (919.6, range 265.6- 1476.6) CXXC5 expression analyzed with the PCR assay.

Supplementary Table 5.

Global gene expression analyses of patients with high and low expression of CXXC5 - genes showing increased expression in patients with low CXXC5 expression. We investigated the global gene expression profile for 48 consecutive/unselected patients and thereafter we compared the profiles for the 15 patients with the highest and the 15 patients with the lowest CXXC5 expression. The table gives the genes with an increased expression in patients with low CXXC5 levels, i.e. a p-value corresponding to <0.05 and a false discovery rate of 1.0.

Upregulated in Low RINF				Score	Fold Change
32812	VCAN	1462	[java.awt.Color[r=0,g=0,b=0]]	3.21221611731678E-7	-15.613263207131403
3476	CD14	929	[java.awt.Color[r=0,g=0,b=0]]	1.628050144536443E-6	-14.306416067436633
25003	NCF1	653361	[java.awt.Color[r=0,g=0,b=0]]	2.563520821882071E-10	-13.06689404061632
8518	HK3	3101	[java.awt.Color[r=0,g=0,b=0]]	1.3480964511195502E-15	-10.895285589441226
28888	S100A12	6283	[java.awt.Color[r=0,g=0,b=0]]	1.927634588300091E-7	-10.851049079788002
24622	MS4A6A	64231	[java.awt.Color[r=0,g=0,b=0]]	6.329540942371881E-10	-10.511810996941314
23770	MAFB	9935	[java.awt.Color[r=0,g=0,b=0]]	1.3608225574805158E-8	-10.256270833966898
4989	DEFB1	1672	[java.awt.Color[r=0,g=0,b=0]]	9.528903575849452E-6	-10.129249672798741
6678	FGL2	10875	[java.awt.Color[r=0,g=0,b=0]]	5.974815763007975E-9	-9.987392241447596
7237	FPR1	2357	[java.awt.Color[r=0,g=0,b=0]]	1.323181842646285E-8	-9.477584984955444
6576	FCER1G	2207	[java.awt.Color[r=0,g=0,b=0]]	2.3042461028655925E-10	-9.456219035174836
879	AQP9	366	[java.awt.Color[r=0,g=0,b=0]]	6.320589563992989E-9	-9.112205480657286
25005	NCF1C	654817	[java.awt.Color[r=0,g=0,b=0]]	3.307140050080916E-10	-9.083633714946007
28455	RNASE2	6036	[java.awt.Color[r=0,g=0,b=0]]	8.40191799116475E-7	-9.035894018233183
2188	C19ORF59	199675	[java.awt.Color[r=0,g=0,b=0]]	7.013910279886439E-9	-8.910010930445614
6593	FCN1	2219	[java.awt.Color[r=0,g=0,b=0]]	4.9880621452112105E-5	-8.312830055270666
12174	IFI30	10437	[java.awt.Color[r=0,g=0,b=0]]	5.9146355356831166E-9	-8.158146125044372
29077	SCPEP1	59342	[java.awt.Color[r=0,g=0,b=0]]	4.611696890264615E-10	-8.098113066531155
28456	RNASE3	6037	[java.awt.Color[r=0,g=0,b=0]]	1.295931066530717E-6	-8.074749288328936
1445	BCL6	604	[java.awt.Color[r=0,g=0,b=0]]	1.528244071309346E-13	-8.053050653939955
6828	FLJ22662	79887	[java.awt.Color[r=0,g=0,b=0]]	2.3561682395858453E-6	-7.848701063743838
28901	S100A9	6280	[java.awt.Color[r=0,g=0,b=0]]	4.829708417381405E-6	-7.4245945331767755
29248	SERPINA1	5265	[java.awt.Color[r=0,g=0,b=0]]	2.2745387035030291E-7	-7.329014439723435
23740	LYZ	4069	[java.awt.Color[r=0,g=0,b=0]]	2.8209074938792313E-8	-7.137108973320186
29883	SLC7A7	9056	[java.awt.Color[r=0,g=0,b=0]]	1.6427057480978583E-7	-7.043171026109201
756	ANXA5	308	[java.awt.Color[r=0,g=0,b=0]]	9.3787476868166E-8	-6.909155589277855
6679	FGR	2268	[java.awt.Color[r=0,g=0,b=0]]	1.577261914264469E-13	-6.865838795246542
29557	SLC11A1	6556	[java.awt.Color[r=0,g=0,b=0]]	7.895815969150579E-10	-6.805699132806284
4715	CYBB	1536	[java.awt.Color[r=0,g=0,b=0]]	1.6097639322509046E-8	-6.77480113037592
3479	CD163	9332	[java.awt.Color[r=0,g=0,b=0]]	7.51008090893673E-7	-6.742969990057105
24427	MNDA	4332	[java.awt.Color[r=0,g=0,b=0]]	5.053086262542702E-8	-6.602151373845691
3560	CD93	22918	[java.awt.Color[r=0,g=0,b=0]]	6.50037333544827E-6	-6.469679772553619
4601	CTSH	1512	[java.awt.Color[r=0,g=0,b=0]]	7.865054207496005E-8	-6.411394094444394
24624	MS4A7	58475	[java.awt.Color[r=0,g=0,b=0]]	3.577834798168954E-11	-6.342648861577022
25006	NCF2	4688	[java.awt.Color[r=0,g=0,b=0]]	1.196202583008517E-10	-6.3321804531869965
28904	S100P	6286	[java.awt.Color[r=0,g=0,b=0]]	1.4750694003728701E-5	-6.307978045827886
27921	RAB31	11031	[java.awt.Color[r=0,g=0,b=0]]	3.163145955313928E-6	-6.129421226649932
28900	S100A8	6279	[java.awt.Color[r=0,g=0,b=0]]	1.0464759643060944E-5	-6.079357946225208
13573	LILRA5	353514	[java.awt.Color[r=0,g=0,b=0]]	3.4323658863740955E-9	-5.978075601720718
23711	LY96	23643	[java.awt.Color[r=0,g=0,b=0]]	2.5771031493580954E-7	-5.9582499350189435
13571	LILRA3	11026	[java.awt.Color[r=0,g=0,b=0]]	3.922034722813991E-6	-5.917820838357176
3715	CEBPD	1052	[java.awt.Color[r=0,g=0,b=0]]	1.100273745762623E-5	-5.866634262422429
4603	CTSL1	1514	[java.awt.Color[r=0,g=0,b=0]]	4.878748878500179E-9	-5.799099309864241
3520	CD36	948	[java.awt.Color[r=0,g=0,b=0]]	1.1850935548665708E-5	-5.724643729437547
28154	RBM47	54502	[java.awt.Color[r=0,g=0,b=0]]	2.9721652018989966E-11	-5.68442253759167
3924	CHST15	51363	[java.awt.Color[r=0,g=0,b=0]]	1.6698697325263855E-7	-5.597742893988707
32863	VNN2	8875	[java.awt.Color[r=0,g=0,b=0]]	3.2944542336275754E-8	-5.572213742877731
3489	CD1D	912	[java.awt.Color[r=0,g=0,b=0]]	2.8711382547861956E-10	-5.558472338284392
8077	GRN	2896	[java.awt.Color[r=0,g=0,b=0]]	5.5202066083059937E-11	-5.55676341403172
2681	C5AR1	728	[java.awt.Color[r=0,g=0,b=0]]	4.996115383776097E-8	-5.498487990085403

Supplementary Table 6.

Global gene expression analyses of patients with high and low expression of CXXC5 - genes showing increased expression in patients with high CXXC5 expression. We investigated the global gene expression profile for 48 consecutive/unselected patients and thereafter we compared the profiles for the 15 patients with the highest and the 15 patients with the lowest CXXC5 expression. The table gives the genes with an increased expression in patients with high CXXC5 levels, i.e. a p-value corresponding to <0.05 and a false discovery rate of 1.0.

Upregulated in high RINF				Score	Fold Change
2836	C7ORF41	222166	[java.awt.Color[r=0,g=0,b=0]]	7.993997888212845E-5	2.7168730858634866
13080	KIT	3815	[java.awt.Color[r=0,g=0,b=0]]	0.0010643759632312812	2.721945394817092
25216	NGFRAP1	27018	[java.awt.Color[r=0,g=0,b=0]]	8.75893630719354E-4	2.7368375144456447
23801	MAGED1	9500	[java.awt.Color[r=0,g=0,b=0]]	3.5298407188277035E-7	2.754221070160793
6364	FAM69B	138311	[java.awt.Color[r=0,g=0,b=0]]	3.841791613598621E-5	2.755431030497114
12746	KCNK17	89822	[java.awt.Color[r=0,g=0,b=0]]	7.520845104702447E-6	2.7649986928375947
4800	CYTL1	54360	[java.awt.Color[r=0,g=0,b=0]]	0.013294194756241422	2.772726358377048
4107	CMBL	134147	[java.awt.Color[r=0,g=0,b=0]]	8.17000066157049E-6	2.7748061056023583
24148	MEX3B	84206	[java.awt.Color[r=0,g=0,b=0]]	6.161353745298043E-7	2.7881166656240923
8210	H1F0	3005	[java.awt.Color[r=0,g=0,b=0]]	0.025819626898921873	2.8184900589727047
3235	CCDC102A	92922	[java.awt.Color[r=0,g=0,b=0]]	1.6758249084834407E-13	2.829036024977644
9520	HS.355933		[java.awt.Color[r=0,g=0,b=0]]	3.86536292635464E-8	2.842272706411284
7277	FSCN1	6624	[java.awt.Color[r=0,g=0,b=0]]	4.8171748230028206E-4	2.85682586809982
31507	TM4SF1	4071	[java.awt.Color[r=0,g=0,b=0]]	0.008981286452042771	2.857637646374968
11433	HS.571502		[java.awt.Color[r=0,g=0,b=0]]	2.1782580363444813E-4	2.868755664391878
13401	LAPTM4B	55353	[java.awt.Color[r=0,g=0,b=0]]	0.0029987511840626822	2.9421118983972194
25658	OCIAD2	132299	[java.awt.Color[r=0,g=0,b=0]]	4.455986001101957E-10	2.9429433717774534
29215	SEPP1	6414	[java.awt.Color[r=0,g=0,b=0]]	0.004796390436971436	2.9437406371471058
17829	LOC642299	642299	[java.awt.Color[r=0,g=0,b=0]]	5.683084454198753E-6	2.983931004441744
23826	MAMDC2	256691	[java.awt.Color[r=0,g=0,b=0]]	0.017899182254313094	2.9972621669822574
2687	C5ORF23	79614	[java.awt.Color[r=0,g=0,b=0]]	1.0526008966222872E-5	3.1196380589548265
1189	ATP1B1	481	[java.awt.Color[r=0,g=0,b=0]]	3.5654987608912876E-6	3.1359897024818237
7823	GOLGA8B	440270	[java.awt.Color[r=0,g=0,b=0]]	6.193808657587966E-5	3.1568659010340046
5873	EPDR1	54749	[java.awt.Color[r=0,g=0,b=0]]	1.211444468569047E-8	3.1951762892122346
24374	MLLT11	10962	[java.awt.Color[r=0,g=0,b=0]]	1.935381000913912E-6	3.2471477642847484
12587	ITM2A	9452	[java.awt.Color[r=0,g=0,b=0]]	1.635458112063708E-4	3.346194233561899
629	ANGPT1	284	[java.awt.Color[r=0,g=0,b=0]]	0.0013748998991857506	3.3699551338450178
239	ADA	100	[java.awt.Color[r=0,g=0,b=0]]	6.742451691120886E-7	3.370668804404996
7495	GATA2	2624	[java.awt.Color[r=0,g=0,b=0]]	5.884791027875886E-7	3.3852034325649796
3655	CDK6	1021	[java.awt.Color[r=0,g=0,b=0]]	1.4587365621044463E-8	3.404359479219713
30397	SOCS2	8835	[java.awt.Color[r=0,g=0,b=0]]	0.003908763733115651	3.4448060235342557
4215	COL24A1	255631	[java.awt.Color[r=0,g=0,b=0]]	2.911476683772374E-6	3.4534504878981958
7972	GPR56	9289	[java.awt.Color[r=0,g=0,b=0]]	2.4592216840708994E-5	3.6064507905885055
2619	C3ORF54	389119	[java.awt.Color[r=0,g=0,b=0]]	1.0436377472310969E-8	3.6357665767829936
31341	TFPI	7035	[java.awt.Color[r=0,g=0,b=0]]	1.1748862559797107E-7	3.7066411015771696
32199	TSC22D1	8848	[java.awt.Color[r=0,g=0,b=0]]	2.2240912964742834E-7	3.760890298819047
12589	ITM2C	81618	[java.awt.Color[r=0,g=0,b=0]]	2.9906675520559737E-6	3.8420076449029317
30437	SOX4	6659	[java.awt.Color[r=0,g=0,b=0]]	2.514154430769112E-8	3.849372228156155
27611	PRSSL1	400668	[java.awt.Color[r=0,g=0,b=0]]	0.0010605725082625072	3.8728167076118054
4698	CXXC5	51523	[java.awt.Color[r=0,g=0,b=0]]	2.01088282769902E-10	3.9250864717974956
3519	CD34	947	[java.awt.Color[r=0,g=0,b=0]]	0.0010246913481842012	3.9797638812398892
24795	MYCN	4613	[java.awt.Color[r=0,g=0,b=0]]	4.0591868489034405E-4	4.00173916881887
17557	LOC442597	442597	[java.awt.Color[r=0,g=0,b=0]]	2.7008721173037734E-6	4.1530426887533745
8177	GUCY1A3	2982	[java.awt.Color[r=0,g=0,b=0]]	4.205638831787519E-8	4.525713773773244
12837	KIAA0125	9834	[java.awt.Color[r=0,g=0,b=0]]	2.3001401307775045E-5	4.5278333644031825
6299	FAM30A	29064	[java.awt.Color[r=0,g=0,b=0]]	3.004750117249858E-6	4.610662953161176
486	AKR1C3	8644	[java.awt.Color[r=0,g=0,b=0]]	3.4373850264788465E-7	4.89743516230991
4327	CPA3	1359	[java.awt.Color[r=0,g=0,b=0]]	4.1178808832263453E-4	5.483576405315826
16256	LOC284422	284422	[java.awt.Color[r=0,g=0,b=0]]	7.054697379703632E-8	5.53781489775216
5351	DNTT	1791	[java.awt.Color[r=0,g=0,b=0]]	0.005845631217744377	5.589516733979638

<u>Supplementary Table 7.</u> Effects of CXXC5 knockdown on gene expression in AML cell lines. CXXC5 was knocked down by lentiviral transfection of shCXXC5, and the global gene expression profile was examined by using mRNA microarray analysis. Identified genes were then compared with those genes showing the largest difference when comparing AML patients with high and low CXXC5 expression. The table gives the genes that showed at least a 2-fold alteration (i.e. comparable to the downregulation of CXXC5) in one cell line or at least a 1.5 fold alteration for at least two cell lines (↑, upregulation).

**Gene id**	**Name**	**Effect of CXXC5 knockdown**	**Relevance of the gene/protein to carcinogenesis and chemosensitivity**	**Cell line examined**
TSC22D1	TSC22 domain family, member 1	↑	This is a transcriptional regulator, its promoter shows binding sites for several transcription factors, the gene can thus integrate multiple signaling events, and it is important during embryogenesis both in Drosophilia and mouse [1]. It can contribute to induction of apoptosis in gastric and breast cancer cells, increase the sensitivity to different anticancer drugs [1] and enhance radiation sensitivity [2]. TSC22 is a potential tumor suppressor in human AML [3]. Its expression seems to be controlled by FLT3-initiated signaling; forced expression has growth-inhibitory effects and induces adhesiveness as well as monocytic differentiation.	MV4-11 (x1.5) K562 (x1.5)
SEPP1	Selenoprotein P.plasma.1	↑	The gene encodes a secreted glycoportein that contains most of the plasma selenium [4]. Selenium is involved in the regulation of proliferation/cell cycle progression and apoptosis, especially supranutritional doses and certain metabolites have growth-inhibitory effects [5].	MV4-11 (x2) UT7 (x1.5)
KIT/CD117	v-kit Hardy-Zuckermann 4 seline sarcoma viral oncogene homolog	↑	CD117 expression is detected for a major part of AML patients, the expression does not have any prognostic impact in patients receiving intensive chemotherapy but rather seems to be a marker of differentiation [6]. The ligand Stem Cell Factor is a growth factor for primary AML cells for most patients [7, 8].	K562 (x2)
ANGPT1	Angiopoietin 1	↑	This is a proangiogenic cytokine constitutively released by primary human AML cells for most patients [9-12]. Angiopoietin 1 and angiopoietin 2 both bind to the Tie2 receptor and depending on the biological function Angiopoetin 2 can function as a natural antagonist of Angiopoietin 1 [13]. Angiopoietin 2 levels have a prognostic impact in human AML, whereas no studies have shown any independent prognostic impact of agonistic Angiopoietin 1. An adverse prognostic impact with increased relapse risk was seen in all 3 studies investigating blood or bone marrow plasma levels [14-16] as well as in one study investigating gene expression in marrow blasts [17], whereas one histochemocal study [18] and one gene expression study [19] showed a good prognostic impact. Increased Angiopoietin 1 levels may thus antagonize adverse effects of Angiopoietin 2 in AML cells.	UT7 (x2)

References

1. Kawamata H, Fujimori T and Imai Y. TSC-22 (TGF-beta stimulated clone-22): a novel molecular target for differentiation-inducing therapy in salivary gland cancer. Curr Cancer Drug Targets. 2004; 4(6):521-529.
2. Hino S, Kawamata H, Omotehara F, Uchida D, Miwa Y, Begum NM, Yoshida H, Sato M and Fujimori T. Cytoplasmic TSC-22 (transforming growth factor-beta-stimulated clone-22) markedly enhances the radiation sensitivity of salivary gland cancer cells. Biochem Biophys Res Commun. 2002; 292(4):957-963.
3. Lu Y, Kitaura J, Oki T, Komeno Y, Ozaki K, Kiyono M, Kumagai H, Nakajima H, Nosaka T, Aburatani H and Kitamura T. Identification of TSC-22 as a potential tumor suppressor that is upregulated by Flt3-D835V but not Flt3-ITD. Leukemia. 2007; 21(11):2246-2257.
4. Burk RF and Hill KE. Selenoprotein P-expression, functions, and roles in mammals. Biochim Biophys Acta. 2009; 1790(11):1441-1447.
5. Zeng H. Selenium as an essential micronutrient: roles in cell cycle and apoptosis. Molecules. 2009; 14(3):1263-1278.
6. Schwartz S, Heinecke A, Zimmermann M, Creutzig U, Schoch C, Harbott J, Fonatsch C, Loffler H, Buchner T, Ludwig WD and Thiel E. Expression of the C-kit receptor (CD117) is a feature of almost all subtypes of de novo acute myeloblastic leukemia (AML), including cytogenetically good-risk AML, and lacks prognostic significance. Leuk Lymphoma. 1999; 34(1-2):85-94.
7. Bruserud O, Gjertsen BT, Foss B and Huang TS. New strategies in the treatment of acute myelogenous leukemia (AML): in vitro culture of aml cells--the present use in experimental studies and the possible importance for future therapeutic approaches. Stem Cells. 2001; 19(1):1-11.
8. Reikvam H, Oyan AM, Kalland KH, Hovland R, Hatfield KJ and Bruserud O. Differences in proliferative capacity of primary human acute myelogenous leukaemia cells are associated with altered gene expression profiles and can be used for subclassification of patients. Cell proliferation. 2013; 46(5):554-562.
9. Hatfield KJ, Hovland R, Oyan AM, Kalland KH, Ryningen A, Gjertsen BT and Bruserud O. Release of angiopoietin-1 by primary human acute myelogenous leukemia cells is associated with mutations of nucleophosmin, increased by bone marrow stromal cells and possibly antagonized by high systemic angiopoietin-2 levels. Leukemia. 2008; 22(2):287-293.
10. Reikvam H, Hatfield KJ, Lassalle P, Kittang AO, Ersvaer E and Bruserud O. Targeting the angiopoietin (Ang)/Tie-2 pathway in the crosstalk between acute myeloid leukaemia and endothelial cells: studies of Tie-2 blocking antibodies, exogenous Ang-2 and inhibition of constitutive agonistic Ang-1 release. Expert Opin Investig Drugs. 2010; 19(2):169-183.
11. Reikvam H, Hatfield KJ, Fredly H, Nepstad I, Mosevoll KA and Bruserud O. The angioregulatory cytokine network in human acute myeloid leukemia - from leukemogenesis via remission induction to stem cell transplantation. Eur Cytokine Netw. 2012; 23(4):140-153.
12. Hatfield KJ, Olsnes AM, Gjertsen BT and Bruserud O. Antiangiogenic therapy in acute myelogenous leukemia: targeting of vascular endothelial growth factor and interleukin 8 as possible antileukemic strategies. Curr Cancer Drug Targets. 2005; 5(4):229-248.

13. Wong AL, Haroon ZA, Werner S, Dewhirst MW, Greenberg CS and Peters KG. Tie2 expression and phosphorylation in angiogenic and quiescent adult tissues. Circ Res. 1997; 81(4):567-574.

14. Kümpers P, Koenecke C, Hecker H, Hellpap J, Horn R, Verhagen W, Buchholz S, Hertenstein B, Krauter J, Eder M, David S, Gohring G, Haller H and Ganser A. Angiopoietin-2 predicts disease-free survival after allogeneic stem cell transplantation in patients with high-risk myeloid malignancies. Blood. 2008; 112(5):2139-2148.

15. Lee CY, Tien HF, Hu CY, Chou WC and Lin LI. Marrow angiogenesis-associated factors as prognostic biomarkers in patients with acute myelogenous leukaemia. Br J Cancer. 2007; 97(7):877-882.

16. Schliemann C, Bieker R, Thoennissen N, Gerss J, Liersch R, Kessler T, Buchner T, Berdel WE and Mesters RM. Circulating angiopoietin-2 is a strong prognostic factor in acute myeloid leukemia. Leukemia. 2007; 21(9):1901-1906.

17. Hou HA, Chou WC, Lin LI, Tang JL, Tseng MH, Huang CF, Yao M, Chen CY, Tsay W and Tien HF. Expression of angiopoietins and vascular endothelial growth factors and their clinical significance in acute myeloid leukemia. Leuk Res. 2008; 32(6):904-912.

18. Schliemann C, Bieker R, Padro T, Kessler T, Hintelmann H, Buchner T, Berdel WE and Mesters RM. Expression of angiopoietins and their receptor Tie2 in the bone marrow of patients with acute myeloid leukemia. Haematologica. 2006; 91(9):1203-1211.

19. Loges S, Heil G, Bruweleit M, Schoder V, Butzal M, Fischer U, Gehling UM, Schuch G, Hossfeld DK and Fiedler W. Analysis of concerted expression of angiogenic growth factors in acute myeloid leukemia: expression of angiopoietin-2 represents an independent prognostic factor for overall survival. J Clin Oncol. 2005; 23(6):1109-1117.
